# Effectiveness of Transitional Interventions in Improving Patient Outcomes and Service Use After Discharge From Psychiatric Inpatient Care: A Systematic Review and Meta-Analysis

**DOI:** 10.3389/fpsyt.2019.00969

**Published:** 2020-01-21

**Authors:** Anna Hegedüs, Bernd Kozel, Dirk Richter, Johann Behrens

**Affiliations:** ^1^ Research Institute, Careum School of Health Ltd., Zurich, Switzerland; ^2^ International Graduate Academy (InGrA), “Participation as Goal of Nursing and Therapy”, Faculty of Medicine, Institute of Health and Nursing Sciences, Martin-Luther-University Halle-Wittenberg, Halle-Wittenberg, Germany; ^3^ Department of Clinical Nursing Science, University Psychiatric Hospital of Bern, Bern, Switzerland; ^4^ Centre for Psychiatric Rehabilitation, University Psychiatric Hospital of Bern, Bern, Switzerland; ^5^ Department of Health Professions, Bern University of Applied Sciences, Bern, Switzerland; ^6^ Faculty of Medicine, Institute of Health and Nursing Sciences, Martin-Luther-University Halle-Wittenberg, Halle-Wittenberg, Germany; ^7^ Frankfurt Institute of Supervision, Institutional Analysis and Social Research (ISIS non profit e.V.), Frankfurt, Germany

**Keywords:** systematic review, meta-analysis, discharge, transitional care, psychiatry, mental health

## Abstract

**Background:** The transition from psychiatric hospital to community is often hindered by challenges that influence community adjustment and continuity of care. Transitional interventions with bridging components are provided prior to discharge and continue beyond inpatient care. They provide continuity of care and may be effective in preventing readmission. We aimed to assess the effectiveness of transitional interventions with predischarge and postdischarge components in reducing readmissions and improving health-related or social outcomes of patients discharged from psychiatric hospitals.

**Methods:** We conducted a systematic review by searching electronic databases (MEDLINE, Embase, Cochrane Library, CINAHL, PsycINFO, and Psyndex) and included randomized, nonrandomized, and one-group study designs. A random effects meta-analysis was conducted with randomized controlled trials (RCTs) reporting data on readmission rates. Other study designs were synthesized qualitatively.

**Results:** After screening 2,673 publications, 16 studies (10 RCTs, three quasi-experimental, and three cohort studies) were included and nine RCTs were included in the meta-analysis. The tested interventions included components from case management, psychoeducation, cognitive behavioral therapy, and peer support. All studies with significant improvements in at least one outcome provided elements of case management, most frequently in combination with cognitive behavioral therapy and psychoeducation. Readmission rates during follow-up ranged between 13% and 63% in intervention groups and 19% and 69% in control groups. Overall, we found an odds ratio of 0.76 (95% confidence interval = 0.55–1.05) for readmission due to transitional interventions. Heterogeneity was low at only 31% (p = 0.17) and the funnel plot indicated no obvious publication biases.

**Conclusions:** We observed that transitional interventions with bridging components were no more effective in reducing readmission than treatment as usual; however, these results are based on limited evidence. Therefore, additional high-quality research is required to conclude the effectiveness of transitional interventions. Nevertheless, transitional interventions with bridging components are preferred by service users and could be an alternative to strategies regularly employed.

## Introduction

For psychiatric patients, the transition from hospital to community is often hindered by challenges that influence community adjustment and continuity of care (1). The first days and weeks after discharge from psychiatric institutions represent a critical phase for patients. During this time period, difficulties often arise in everyday life, such as increased risk of suicide, craving, anxiety, loneliness, lack of self-esteem, stigmatization, lack of treatment adherence, and difficulties in coping with recurring symptoms (2–13). Any of these challenges can result in symptom relapse or readmission to inpatient care. Readmission rates after 30 days vary between 9% and 15% in Canada and Europe (14, 15), and are approximately 18% within 4 months in Canada (14), 13% within 6 months in the United States (16), and between 33% and 48% within 12 months in Europe or New Zealand (15, 17). Across all countries, the risk of readmission is highest in the first 30 days after discharge (15, 18). Therefore, preventive measures are recommended to ensure the availability of seamless transition from inpatient to community care (15).

Interventions that are provided prior to discharge and continued beyond inpatient care are referred to as transitional interventions with bridging components (7). They are structured discharge management strategies and allow for the maintenance of therapeutic relationships that are established during inpatient stays. By offering support before and beyond the inpatient stay, healthcare professionals can emphasize care needs before discharge and address or follow-up with those needs in the community setting. This combination of elements prior to and after discharge fulfils patients' desire for continuity of care (19) and is considered helpful (20) and promising in supporting the effective transition from hospital to community.

Interventions aiming to improve the transition from hospital to home have been tested in studies and partly summarized in systematic reviews. These systematic reviews have either focused on a wide range of interventions, including preintervention and postintervention components (7, 21), specific patient groups (22), and settings or services (23, 24). For example, Vigod et al. (7) found evidence from nonrandomized trials indicating that psychoeducational interventions help to improve disease management and everyday skills, which may reduce readmission rates of psychiatric patients. Alternatively, discharge interventions for patients with severe depression demonstrated no significant effects on readmission rates or the improvement of depressive symptoms (22); however, patients have reported that discharge planning and follow-up after discharge are essential to prevent readmission (15).

The effectiveness of transitional interventions with bridging components has not yet been summarized in systematic reviews. By limiting the interventions to those with bridging components, we expect better homogeneity and comparability than in previous systematic reviews. Therefore, we conducted a systematic review and meta-analysis to assess the effectiveness of transitional interventions with predischarge and postdischarge components in reducing readmissions and improving health-related or social outcomes of patients discharged from psychiatric hospitals.

## Methods

The present systematic review and meta-analysis adheres to PRISMA guidelines (25). A review protocol was publicly registered on PROSPERO (registration no.: CRD42019122456).

### Search Strategy

We searched the following databases: MEDLINE, Embase, Cochrane Library, CINAHL, PsycINFO, and Psyndex. Documents published between 1998 and May 31, 2018 were included in our search strategy. Database-specific searches included the following index terms: (“bridging” OR “transitional care” OR “patient discharge” OR “discharge planning”) AND (“Psychiatric Hospital” OR “Mental Institution” OR “Mental Hospital”) AND (“intervention” OR “programme” OR “preparation”) NOT (child* OR dement*). Additional searches were conducted in Google and Google Scholar to find relevant grey literature. Hand searching of the references of key papers (e.g., existing systematic reviews and included articles) complemented the search. The detailed search strategy is available upon reasonable request.

### Inclusion/Exclusion Criteria

To be included, studies had to meet the following criteria: published studies or study protocols written in German or English, participants aged 18-65 years, participants had a psychiatric diagnosis and were discharged from a psychiatric inpatient unit. Included interventions were those that aim to improve discharge from psychiatric inpatient care to home with a combination of predischarge and postdischarge components (e.g., need assessment or development of discharge plan predischarge and home visits or telephone contacts postdischarge). Moreover, all components of the intervention must have emanated from the inpatient setting. Finally, although randomization is desirable to minimize selection bias, it may not be feasible in mental health care. We therefore included nonrandomized or one-group study designs into our qualitative synthesis.

The following exclusion criteria were defined: interventions or programs on a structural or organizational level; psychotherapeutic treatment programs with a specific focus (e.g., therapy for substance dependence or medication adherence); interventions specifically targeting homeless persons; participants with physical or mental handicap; and discharge from forensic settings.

### Data Extraction and Risk of Bias Assessment

Titles and abstracts of studies retrieved from database searches and additional sources were independently screened by two authors (AH and BK) to identify those that met inclusion criteria. The full texts were then retrieved and independently assessed for eligibility by the same two authors. Disagreements over the eligibility of any studies were resolved through discussion.

Data extraction from the included studies was carried out by one team member (AH) and checked for accuracy by another (BK). Extracted information included: study design, setting, and population; details of the intervention and control conditions; recruitment and study completion rates; outcomes and times of measurement; and information for assessment of the risk of bias. Missing or nonreported data on the primary outcome (readmission rates) was requested from study authors.

To classify the intensity of the interventions, we adapted the intervention intensity score developed by Holzinger et al. (22). The score (low/moderate/high intensity) considers the length of intervention, number of intervention components (e.g., psychoeducation, cognitive behavioral therapy [CBT], case management, and peer support), and number of contacts with the patient. Details of our adaptations and ratings are available from AH.

Two authors (AH and BK) independently assessed the risk of bias in the included studies by using the Revised Cochrane Risk-of-Bias for randomized trials (26) and the Mixed Methods Appraisal Tool for all other study designs (27). Discrepancies were solved by discussion within the team.

### Data Analysis

Studies were included in the present meta-analysis if data were available on readmission rates in absolute numbers or percentages. The random effects meta-analysis was conducted with the package ‘meta' (version 4.9-5) in R statistical software (version 3.6.1; R Core Team, Vienna, Austria). Odds ratios were calculated for effect sizes. Study heterogeneity was assessed by I^2^, and 95% confidence intervals (CIs) were used to assess uncertainty. Forest and funnel plots were used for the graphical display of effect sizes and analyzing publication bias, respectively. Subgroup comparisons were conducted for length of intervention, risk of bias, and intervention intensity.

Meta-analyses of other outcomes were not possible due to the low number of studies and heterogeneous assessment instruments.

## Results

### Study Selection and Characteristics

**Figure 1:**
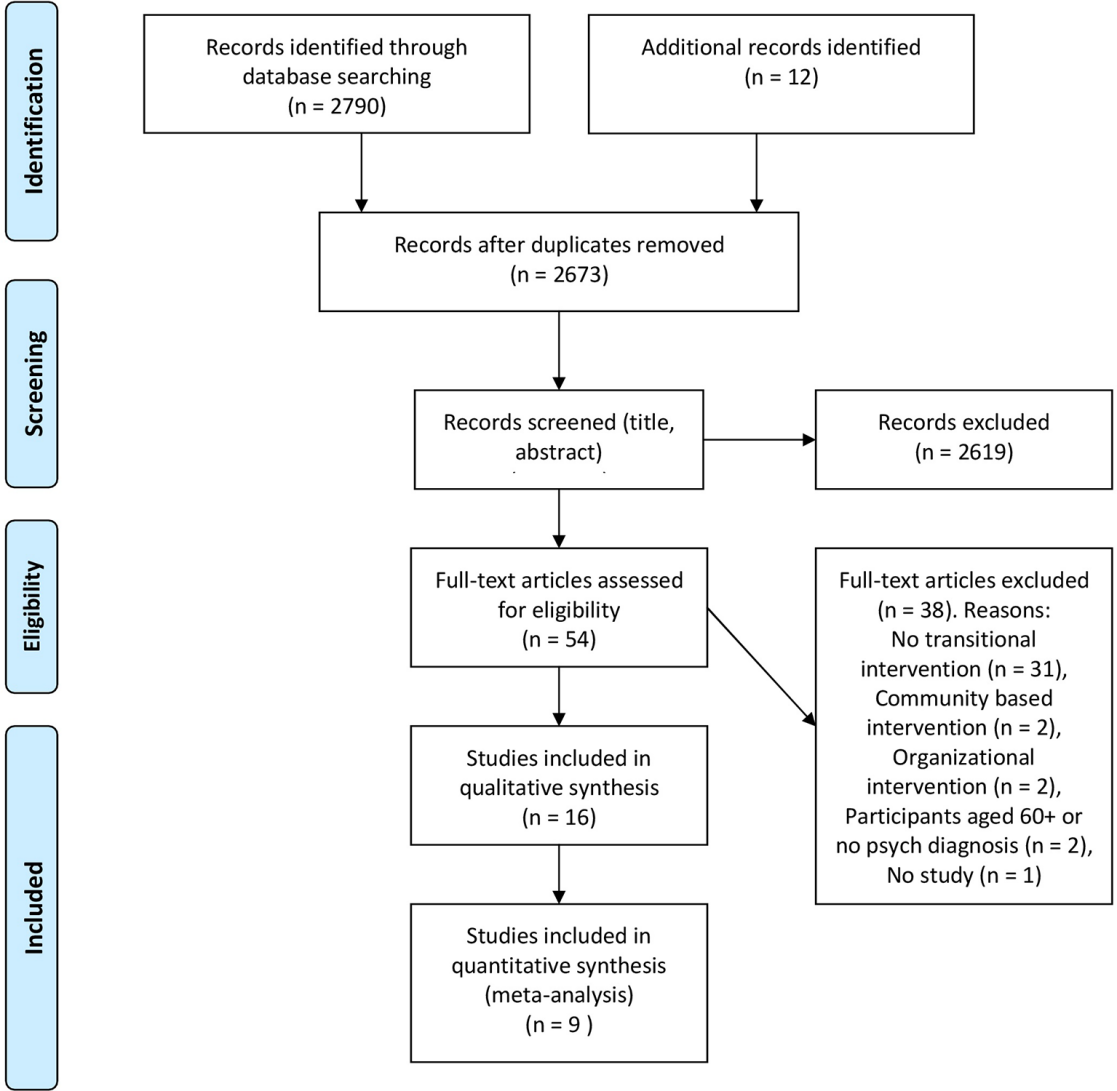
Flow diagram of study selection process.

The search strategy yielded 2,673 publications, which after screening resulted in 16 included studies. There were 10 RCTs, three quasi-experimental studies, and three cohort studies. From this, nine RCTs were included in the meta-analysis (**Figure 1**). In the RCTs, the percentage of participants with a diagnosis of psychosis or schizophrenia varied between 8% and 73% and between 26% and 75% for participants with depressive or anxiety disorders. In the cohort studies, a strong focus on patients with psychosis or schizophrenia was apparent (range 87%-100%). Study characteristics are shown in **Table 1**.

**Table 1:** Description of included studies.

Reference	Design	Country	Intervention	Intervention Components	Length of intervention (weeks)	Intervention intensity score	Comparison	Initial sample size: IG/CG	Diagnoses of participants		Follow-up
									Psychosis/schizophrenia	Depression/anxiety
Bonsack et al. (28)	RCT	CH	Transitional Case management	CM, CBT	4	++	TAU	51/51	8%	75%	12 months
Dixon et al. (29)	RCT	US	Brief critical time intervention	PE, CM, CBT	12	+++	TAU	64/71	37%	NR	6 months
Forchuk et al. (30)	cRCT	CA	Transitional discharge model	CBT, PS	12	+++	TAU	201/189	48%	42%	12 months
Hanrahan et al. (31)	RCT	US	Transitional Care Model	PE, CM	12	++	TAU	20/20	73%*	75%*	3 months
Hengartner et al. (32)	RCT	CH	Postdischarge Network Coordination Program	CM	12	++	TAU	76/75	27%	34%	18 months
Lay et al. (33)	RCT	CH	Psychoeducation and crisis focused monitoring	PE, CM, CBT	96	+++	TAU	119/119	39%	26%	24 months
Price et al. (34)	RCT	US	Transition to Community program	CM	2	+	TAU	7/6	100%		7 weeks
Puschner et al. (35, 36)	RCT	D	Needs-oriented discharge planning and monitoring for high utilizers	CM	12	+	TAU	241/250	59%	41%	18 months
Reynolds et al. (37)	RCT	UK	Transitional discharge model	CBT, PS	20	++	TAU	11/14	NR	NR	5 months
Simpson et al. (38)	RCT	UK	Peer support for discharged patients	PS	4	+	TAU	20/22	52%	39%	3 months
Cuffel et al. (39)	QE	US	Group 1: Enhanced care management Group 2: intensive care management	CM	12	++	TAU	74**/94***/31	20%	NR	6 months
Hegedüs et al. (40)	QE	CH	Short transitional intervention in psychiatry	CM, CBT	1	+	TAU	20/20	10%	59%	1 week
Khaleghparast et al. (41)	QE	IRN	Discharge planning	CM, CBT	12	++	TAU	23/23	100%		3 months
Batscha et al. (42)	Cohort study	US	Inpatient Transition Intervention by APN transition coach	CM	3	+	-	15	100%		2 weeks
Kidd et al. (43)	Cohort study	CA	Modified Welcome Basket intervention	CM, PS	4	++	-	23	87%	13%	1 month; 6 months readmission
Noda et al. (44)	Cohort study	JPN	Tokyo Musashino Hospital Psychiatric Rehabilitation Service	PE, CM, CBT	52	++	-	224	100%		2, 5, 7 years after discharge

RCT, randomized controlled trial; cRCT, cluster randomized controlled trial; QE, quasi-experimental design; CH, Switzerland; US, United States; CA, Canada; D, Germany; UK, United Kingdom; IRN, Iran; JPN, Japan; Intervention components: PE, psychoeducation, CM, case management, CBT, cognitive behavioral therapy, PS, peer support; TAU, treatment as usual; + low intensity, ++ moderate intensity, +++ high intensity; IG, intervention group; CG, control group; NR, not reported in publication; *multiple diagnoses possible; **enhanced care management, ***intensive care management.

### Description and Classification of Intervention Components

The included studies tested 15 different interventions (**Table 1**). All interventions included multiple components and were conducted by mental health workers, nurses (a portion with a master's degree), case or care managers, social workers, or peer support workers.

Predischarge interventions included components from case management, such as needs assessments (28, 29, 32, 34, 35, 40–43), discharge or care planning (e.g., crisis planning) (28, 31, 35), scheduling or preparing for follow-up appointments (39, 42), and family or carer involvement (28, 44); psychoeducational components, such as individualized psychoeducation (33) and medication reconciliation elements (29, 44); and elements of CBT, such as skills training (29, 40, 41, 44) and peer support (38, 43). Interventions were delivered in one-to-one sessions, except for the study conducted by Khaleghparast et al. (41), where a family member was present and in Noda et al. (44), where skills training was delivered in groups.

Postdischarge components aimed to support patients during a transition period and were most frequently delivered through phone calls, home visits, or letters. Components associated with case management included: efforts to ensure timely follow-up with outpatient care providers (28, 32, 34, 39, 42), treatment coordination (28, 31, 39), timely communication between inpatient staff and outpatient care or community service provider after discharge (29, 34), monitoring of health status or implementation of postdischarge plan (31, 33, 35), and activation of resources in the social network (32). Elements of CBT consisted of therapeutic meetings with staff (30, 37) and skills training (33, 40, 41, 44). In addition, interventions included psychoeducation and counseling (31) or peer support (30, 37, 38, 43). Peer support consisted of facilitating access to local communities, promoting friendship, providing basic necessities, understanding, and encouragement. Similar to the predischarge components, postdischarge contact took place in sessions between patients and mental health workers, occasionally with explicit inclusion of important members of the patient's social network (32).

Interventions lasted between 1 week (40) and 2 years (33), or until a therapeutic relationship was established between the patient and outpatient care provider (30, 37); however, the majority of the interventions ended 3 months after discharge (29, 31, 32, 35, 39, 41).

Control groups received treatment as usual, which included aftercare or treatment planning (39, 40), referral to outpatient treatment (28, 29, 33, 37, 39), arrangements from community mental health services (38), and case and medication management (31). Treatment as usual in the study by Puschner et al. (35) was delivered without a manualized or structured discharge plan. Assistance from inpatient units ended after the patient was discharged, except in the study by Cuffel et al. (39). Finally, three studies did not specify treatment as usual (30, 34, 41) and an additional three studies did not apply a control group design (42–44).

### Risk of Bias

Details on the quality assessment of the included studies are displayed in **Tables 2** and **3**. From the 10 RCTs included, only the study by Hengartner et al. (32) proved to have a low risk of bias. Five RCTs were rated to have some concerns (28, 29, 31, 33, 35) and four studies (30, 34, 37, 38) were rated with high risk of bias. Khaleghparast et al. (41) did not describe a randomization process and was therefore included and rated as a study using a quasi-experimental design. Risk of bias was evident primarily in non-RCTs and studies with a one-group design due to incomplete outcome data (i.e., high drop-out rates) and potential confounders that were not accounted for.

**Table 2:** Quality assessment of randomized controlled studies using the Revised Cochrane Risk-of-Bias tool for randomized trials (26).

Author	Domain 1: randomization process	Domain 2: deviations from intended interventions (assignment)	Domain 3: missing outcome data	Domain 4: measurement of outcome	Domain 5: selection of reported results	Overall judgment
Bonsack et al. (28)	low	low	low	some concerns	low	some concerns
Dixon et al. (29)	low	low	low	low	some concerns	some concerns
Forchuk et al. (30)	low	some concerns	high	low	some concerns	high risk of bias
Hanrahan et al. (31)	low	low	low	some concerns	some concerns	some concerns
Hengartner et al. (32)	low	low	low	low	low	low risk of bias
Lay et al. (33)	low	some concerns	low	low	low	some concerns
Price et al. (34)	some concerns	high	low	low	some concerns	high risk of bias
Puschner et al. (35, 36)	low	low	some concerns	low	low	some concerns
Reynolds et al. (37)	some concerns	some concerns	some concerns	some concerns	some concerns	high risk of bias
Simpson et al. (38)	low	some concerns	some concerns	some concerns	some concerns	high risk of bias

**Table 3:** Quality assessment of nonrandomized studies using the Mixed Methods Appraisal Tool (24).

Quasi-experimental studies	3.1. Are the participants representative of the target population?	3.2. Are measurements appropriate regarding both the outcome and intervention (or exposure)?	3.3. Are there complete outcome data?	3.4. Are the confounders accounted for in the design and analysis?	3.5. During the study period, is the intervention administered (or exposure occurred) as intended?
Cuffel et al. (39)	yes	yes	cannot tell	no	yes
Hegedüs et al. (40)	yes	yes	no	yes	yes
Khaleghparast et al. (41)	yes	yes	yes	no	cannot tell
					
**Cohort Studies**					
Batscha et al. (42)	yes	yes	yes	yes	yes
Kidd et al. (43)	yes	yes	no	no	yes
Noda et al. (44)	yes	yes	yes	no	yes

### Intervention Effects

#### Effects on Readmission

**Figure 2:**
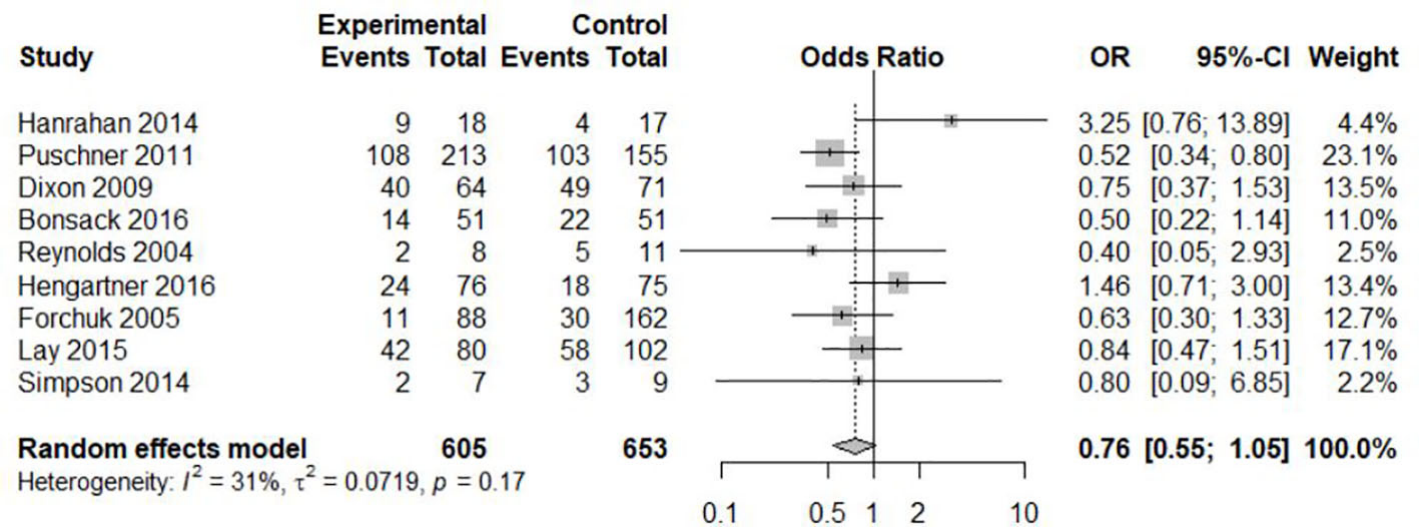
Random effects meta-analysis for readmission.

**Figure 3:**
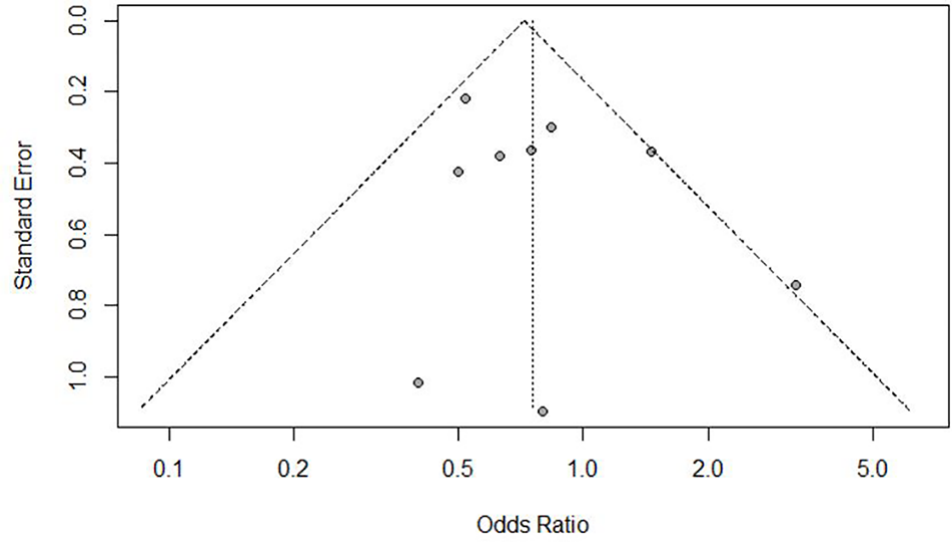
Funnel plot for readmission.

Nine of the included RCTs reported readmission rates (see **Table 4**) and were included in the present meta-analysis. They showed available case data on a total of 1,258 participants (605 in interventional groups, 653 in control). Readmission rates during follow-up ranged between 13% and 63% in intervention groups and 19% and 69% in control groups (treatment as usual). Readmission rates were higher in the control groups in all but two studies (31, 32). Overall, we found an odds ratio of 0.76 (95% CI: 0.55-1.05) for readmission due to transitional interventions (see **Figure 2**). Heterogeneity was low at 31% (p = 0.17). The funnel plot **Figure 3** indicated no obvious publication bias. Finally, subgroup comparisons for risk of bias, duration of intervention, and intensity of intervention found no significant differences (see **Table 5**).

**Table 4:** Overview of study results.

RCT	readmission	service use, inpatient	service use, outpatient	Symptoms	Quality of life	continuity of care	costs	functioning	Satisfaction	hopelessness	loneliness	depression	needs	subjective distress	social support & engagement in community	illness severity	coping	knowledge	difficulty faced after discharge	difficulty of discharge process
Bonsack et al. (28)	=		=																	
Dixon et al. (29)	=	=	(+)	=	=	+			=											
Forchuk et al. (30)	=				=		=													
Hanrahan et al. (31)	=	=			( = )	( = )														
Hengartner et al. (32)	=	=			=			+						-	=	=				
Lay et al. (33)	(+)*	(+)*																		
Price et al. (34)		=				=														
Puschner et al. (35, 36)	=	=	=	=	=		=					=	=							
Reynolds et al. (37)	=			=	=			=												
Simpson et al. (38)	=				=		=			=	=									
																				
**Quasi-experimental studies**																				
Cuffel et al. (39)	=	=	( = )			=														
Hegedüs et al. (40)	=		=														=		=	=
Khaleghparast et al. (41)	+			+														+		
																				
**Cohort studies**																				
Batscha et al. (42)						=														
Kidd et al. (43)	=			=	(+)			+							+					
Noda et al. (44)	+																			

RCT, randomized controlled trial; + significant difference in favor of intervention; = no difference between groups; - significant difference in favor of control; () different measures of domain and results do not coincide; *significant reduction of compulsory readmissions, no change in voluntary admissions.

**Table 5:** Subgroup analysis.

	Odds ratio	95% confidence interval
**Duration of the intervention**		
12 weeks or less (k = 7)	0.778	0.508 – 1.191
13 weeks or more (k = 2)	0.790	0.449 – 1.389
**Risk of bias**		
Low risk (k = 1)	1.462	0.713 – 2.995
Some concerns (k = 5)	0.735	0.444 – 1.216
High risk (k = 3)	0.612	0.315 – 1.189
**Intervention intensity**		
Low (k = 2)	0.528	0.347 – 0.803
Moderate (k = 5)	0.898	0.460 – 1.753
High (k = 2)	0.802	0.509 – 1.261

#### Effects on Patients’ Health-Related or Social Outcomes

Three RCTs, with comparable diagnoses, reported significant improvements favoring the intervention in the following outcomes: compulsory readmission (33), length of compulsory hospital episodes (33), outpatient service use (29), continuity of care (29), and functioning (32) (**Table 4**). The nonrandomized study by Khaleghparast et al. (41) demonstrated significant improvements in all outcome measures (i.e., readmission, symptom severity, and knowledge) in patients with psychosis or schizophrenia. Cohort studies with a one group design showed significant improvements in readmission (44), quality of life (43), functioning (43), social support, and engagement in community (43). All studies with significant effects in at least one outcome provided elements of case management (29, 32, 33, 41, 43, 44), most frequently in combination with CBT and psychoeducation (29, 33, 44) or exclusively CBT (41), or peer support (43).

## Discussion

We conducted a systematic review and meta-analysis of transitional interventions with bridging (predischarge and postdischarge) components to assess their effectiveness in reducing readmission and improving health-related or social outcomes of patients discharged from psychiatric inpatient care. Our quantitative synthesis showed no significant effect of transitional interventions with bridging components on readmission. Considering the limitations of the available studies, there is currently no sufficient evidence to support the effectiveness of transitional interventions in comparison to treatment as usual in the prevention of readmission. Similarly, only individual studies demonstrated significant improvements in health-related and social outcomes; therefore, currently we do not have enough high-quality evidence to highlight the effectiveness of transitional interventions.

Our results corroborate other reviews on discharge interventions that summarize the evidence of a wide range of interventions (7, 21) or focus on either specific patient groups (e.g., depression) (22), settings, or services (e.g., forensic psychiatric services and early discharge) (23, 24). Holzinger et al. (22) found no significant effects of these interventions on readmission; however, included studies showed a tendency toward intervention effectiveness. Similarly, Vigod et al. (7) identified slightly lower readmission rates in the intervention groups of the included studies at 3 and 12 months; however, a quantitative meta-analysis was not possible due to substantial clinical heterogeneity. Steffen et al. (45) identified a significant effect of transitional interventions with predischarge and postdischarge components on readmission. Therefore, by limiting the interventions to those with bridging components, our systematic review adds a unique element to the current research, which can help to further understand some of the mixed results that have been demonstrated thus far. These mixed results could be explained based on the reasons for readmission. Mutschler et al. (46) argue that external factors such as poverty, interpersonal conflicts, and stigma can prevent successful transition to community. The transitional interventions included in our review considered primarily internal factors (e.g., self-efficacy, social or peer support, and coping strategies); external factors were only addressed secondarily (e.g., by peer support workers). Therefore, transitional interventions alone can only partly prevent readmission and improve community integration. Changes at the health and social support level (e.g., comprehensive community care, supported housing, and employment) are important factors to improving community integration of patients discharged from psychiatric inpatient care.

From the patients' point of view, regardless of psychiatric diagnoses or reason for admission, discharge planning, follow-up after discharge, individual coping measures, meaningful activities, and peer support and networks are essential to preventing readmission (15). Our review identified a wide spectrum of interventions and intervention components aiming to meet these needs. Other reviews have concluded that collaborative care interventions, transition managers, and timely communication between inpatient staff and outpatient care or community service providers are crucial bridging components of discharge interventions (7, 22, 47). In our review, we subsumed these components under case management. This was the most frequently used component (13 out of 16 studies) and was used by all studies that showed a significant effect in at least one outcome measure. These elements of case management might contribute to the significant effects of the interventions; however, due to the heterogeneity of the studied interventions and intervention settings, it remains unclear which components affect the outcomes measured. Further research is necessary to address this gap, such as by rigorously defining the interventions and the policy or organizational conditions by which they are applied (e.g., local payment strategies, access to care, and available resources). Researchers are challenged to apply new methods to control for these predefined conditions.

Besides the great variety of interventions, the present review revealed a multitude of outcome measures. In sum, 20 different outcomes were assessed throughout the included studies. Readmission was the most frequently reported outcome and the only one that could be assessed through meta-analysis. This variability in outcome measurements indicates that there is no agreement on outcomes in transitional interventions in psychiatry. Consequently, it is difficult to synthesize and apply the results of different research studies (48). Since the choice of outcome measure is essential for decision making and policy, more emphasis is necessary on the choice of outcome that is suitable for the tested intervention. The development of core outcome sets (i.e., standardized sets of outcomes for a specific clinical area) can provide guidance by including all stakeholders (e.g., service users, caregivers, clinicians, and policy makers) into the development process (48). As a result, these outcomes are valued by the involved parties and account for their interests. In conjunction with the results of a study on the associations between readmission and patient-reported measures in acute psychiatric inpatients (49), we could gain an improved understanding into the relationship between patient experiences, readmission, and patient-reported outcomes. This would also allow for the conduction of meta-analyses on different outcomes and improve the significance of trials for service users, caregivers, and policy makers.

## Conclusions

### Strengths and Limitations

The present review demonstrates clear limitations associated with many of the included publications. Most studies had small sample sizes and were likely underpowered to detect clinically relevant effect sizes. The overall risk of bias was high in four of the 10 RCTs. In addition, the high heterogeneity of intervention components limited their comparability and our ability to generate practice and policy implications. More detailed classification systems are needed (e.g., including characteristics of health/social care system, risk of readmission in certain populations, and quality of inpatient care) to better understand the holistic value of transition interventions.

We limited the included populations by excluding studies where we expected considerable differences during the inpatient stay or unique discharge situations (e.g., long inpatient stays in forensic settings (23, 50) or discharge into homelessness). Nevertheless, the comparability of the included studies was limited by a broad range of interventions and a high number of diverse outcomes and outcome measurements. In addition, we observed diverse definitions of “treatment as usual”, which may limit the significance of the results.

We included studies with participants across various psychiatric diagnostic groups. This did not considerably add to the heterogeneity of the review; however, a quantitative subgroup analysis was not possible. Therefore, future research is recommended to assess the effects of transitional interventions on various psychiatric disorders.

The high risk of bias in the selected RCTs further underlines the limitations of the present study design. Therefore, the synthesis of our present systematic review offers an overview of their efficacy, while not confirming it.

Through rigorous search methods in different databases and hand search of bibliographies, we attempted to identify all possible eligible studies. The funnel plot did not indicate possible limitations regarding publication bias. By including nonrandomized and cohort studies, our present review highlights the value of pragmatic study designs in this setting.

### Conclusions

We observed that transitional interventions with bridging components were not more effective in reducing readmission compared to treatment as usual; however, the results are based on limited evidence. Therefore, we currently cannot make a final recommendation for or against the use of transitional interventions. Nevertheless, transitional interventions with bridging components are preferred by service users and should be considered as alternatives or supplements to regular or no discharge strategies.

Our review highlights that the identification and future design of effective interventions requires higher quality studies with resulting comprehensive publications. First, researchers need to explore proper outcome measures and reach a consensus on measured outcomes (e.g., core outcomes set). This would allow for the conduction of systematic reviews and meta-analyses for health-related and social outcomes, adding to the limited existing evidence. Second, future research on transitional interventions needs more profound classifications of interventions and health care systems. In addition, we need to understand what “treatment as usual” means across various settings to identify effective intervention components and their correlations. Third, studies are needed to assess the effects of transitional interventions or specific intervention components on various disorders. These aspects would allow for the conduction of high-quality research and generate evidence for the best practices.

## Data Availability Statement

All data and material are available upon request from the corresponding author.

## Author Contributions

AH, BK, and JB planned and designed the study. AH and BK conducted the database search, screened studies for inclusion, extracted data, and assessed risk of bias. DR planned and performed the statistical analysis. AH wrote the first draft of the manuscript. All authors contributed to manuscript revision, read, and approved the submitted version.

## Conflict of Interest

The authors declare that the research was conducted in the absence of any commercial or financial relationships that could be construed as a potential conflict of interest.
